# NOX4: A Guilty Party in Stroke Damage

**DOI:** 10.1371/journal.pbio.1000478

**Published:** 2010-09-21

**Authors:** Caitlin Sedwick

**Affiliations:** Freelance Science Writer, San Diego, California, United States of America

**Figure pbio-1000478-g001:**
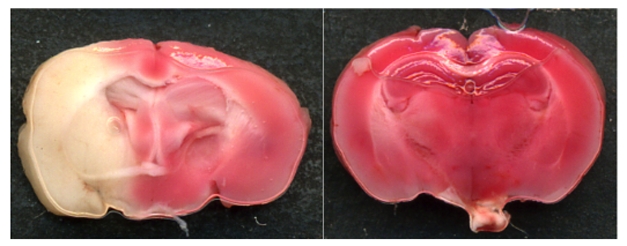
Ischemic infarcts (TTC staining) of brain sections from wild-type mice (left) and mice in which the NOX4 gene has been knocked out (right) 24 hours after a transient occlusion of middle cerebral artery, a model of stroke. Ischemic infarcts (white areas indicating brain damage) are substantially smaller in NOX4 knockout mice and are associated with improved neuronal functions and survival. Image: Christoph Kleinschnitz.

[Fig pbio-1000478-g001]Ischemic stroke is a dangerous medical condition that occurs when blood supply to brain tissues is lost. This usually happens as a result of substantial blood pressure drops, or when blood vessels are blocked by clots. In either case, affected tissues can die, often leading to permanent impairment of mental and physical faculties, the shutdown of essential bodily functions, or even death. If tissue death could be prevented until the blood supply is restored, then stroke prognosis could be improved. However, the mechanisms underlying this damage are still poorly understood, and so far only one effective treatment exists. This treatment, a blood-thinning medication intended to disintegrate blood clots, can't be used in most patients without risk of serious complications. This lack of effective treatments for stroke is becoming ever more urgent as the generation born in the baby boom after WWII are now reaching an age where stroke is of particular concern. Fortunately, a new ray of hope is offered in this issue of *PLoS Biology*, where a group of researchers representing ten institutions and four countries describe a new potential therapeutic target for acute stroke.

Headed by Christoph Kleinschnitz (Germany) and Harald Schmidt (The Netherlands), the group set its sights on the potential for oxidative stress to cause tissue damage after stroke. On a cellular level, oxidative stress occurs when reactive oxygen species (ROS) accumulate within the cell. ROS are normally an important part of cellular physiology, occurring as byproducts of metabolic reactions, or participating in signaling pathways. However, because they are very chemically reactive, they can easily damage DNA, lipids, and proteins when present in high amounts.

Although oxidative stress has long been suspected to play a role in tissue damage after stroke, no study has ever clearly demonstrated a link between the two. Moreover, earlier efforts to prevent ROS-mediated tissue damage by administering antioxidants (which soak up loose ROS) after stroke did not improve therapeutic outcome. Nonetheless, Kleinschnitz and colleagues wondered whether a better outcome might be obtained by blocking ROS production at its source, rather than trying to clean up excess ROS after the fact. This seemed a daunting task because there are many potential ROS sources in the body and brain, but fortunately, a promising lead developed, pointing them toward a gene called NOX4.

Previous studies had shown that NOX4 is a member of a family of proteins that produce ROS in mice. It is found in the cells that line blood vessels throughout the body, but is particularly concentrated in the blood vessels of the brain and in neurons. Additionally, NOX4's expression had been shown to increase when stroke is induced in rats. Kleinschnitz et al. therefore set out to determine how NOX4 contributes to stroke outcome. To do this, they engineered NOX4-deficient mice and explored how these mice cope with stroke.

Strikingly, the group found that NOX4-deficient mice exhibited a smaller extent of tissue death, experienced less severe neurological deficits, and survived for longer after stroke than did wild-type mice. Furthermore, while elevated ROS levels, ROS-damaged proteins, and neuronal cellular suicide were quite evident in the brains of post-stroke wild-type mice, NOX4-deficient mice displayed much lower levels of all these indicators. NOX4 deficiency also correlated with reduced fluid flooding in brain regions adjacent to the stroke, which the authors suggest could be related to decreased damage and leakiness of brain blood vessels.

Importantly, protective effects of NOX4-deficiency are evident in adult mice of both sexes, and also in elderly mice. And, these effects are specific to NOX4 because deficiency in other NOX family members (that are not expressed in the brain) had no effect on the animals' stroke resiliency. These details indicate both the strong specificity and universality of NOX4 deficiency in protecting against stroke-mediated brain damage.

What implications does this work have for therapeutic approaches to stroke? From the perspective of those designing therapeutic interventions, it's encouraging that NOX4-deficient animals appear to do just fine without NOX4; they do not show any signs of dysfunction in other areas where NOX4 is normally expressed (e.g., peripheral blood vessels that serve the kidneys and heart). What's more, Kleinschnitz and colleagues showed that a chemical inhibitor of NOX activity significantly improves post-stroke outcomes in wild-type mice when administered within two hours of stroke onset. A more specific NOX4 inhibitor might therefore turn out to be a useful drug in clinical settings in humans. Future studies will be needed to explore the full potential of these findings.

**Kleinschnitz C, Grund H, Wingler K, Armitage ME, Jones E, et al. (2010) Post-Stroke Inhibition of Induced NADPH Oxidase Type 4 Prevents Oxidative Stress and Neurodegeneration. doi: 10.1371/journal.pbio.1000479**

